# Perceptual Grouping over Time within and across Auditory and Tactile Modalities

**DOI:** 10.1371/journal.pone.0041661

**Published:** 2012-07-26

**Authors:** I-Fan Lin, Makio Kashino

**Affiliations:** NTT Communication Science Laboratories, NTT Corporation, Morinosato, Wakamiya, Japan; Bielefeld University, Germany

## Abstract

In auditory scene analysis, population separation and temporal coherence have been proposed to explain how auditory features are grouped together and streamed over time. The present study investigated whether these two theories can be applied to tactile streaming and whether temporal coherence theory can be applied to crossmodal streaming. The results show that synchrony detection between two tones/taps at different frequencies/locations became difficult when one of the tones/taps was embedded in a perceptual stream. While the taps applied to the same location were streamed over time, the taps applied to different locations were not. This observation suggests that tactile stream formation can be explained by population-separation theory. On the other hand, temporally coherent auditory stimuli at different frequencies were streamed over time, but temporally coherent tactile stimuli applied to different locations were not. When there was within-modality streaming, temporally coherent auditory stimuli and tactile stimuli were not streamed over time, either. This observation suggests the limitation of temporal coherence theory when it is applied to perceptual grouping over time.

## Introduction

In the initial stages of sensory processing, after the photoreceptors on the retina, the hair cells in the cochlea, or the Pacinian corpuscles in the skin are activated, the “features” of stimuli, such as luminance, intensity, and frequency, are extracted and registered in the neural system. In the following processing stages, these spatially or tonotopically distributed features are grouped into coherent objects, segmented from each other and from the background. For visual features, the neural systems can group them together based on Gestalt principles such as similarity, proximity, continuation, closure, symmetry, and common fate. In auditory perception, population-separation theory and temporal coherence theory are proposed to explain how the neural system groups the auditory features [1]. Population-separation theory states that sounds that activate distinct neural populations are heard as separate streams (e.g., [2], [3], [4], [5], [6]). On the other hand, the pure tones that are separated by an octave or more are perceived as a single stream if the tones are fully coherent in time [7]. As a result, temporal coherence theory states that temporally correlated neural activities are combined even when they represent different features, no matter that these features belong to the same modality (e.g., pitch, timber, and location for sounds) or different modalities (e.g., auditory and tactile stimuli).

The somatosensory system has good temporal and spatial resolution. Therefore, it is natural to ask whether the grouping rules found in visual and auditory perception can be applied to tactile perception. To be specific, the present study investigated whether tactile stimuli are segregated if they are applied to different locations (based on population-separation theory) and whether temporally coherent tactile stimuli that are applied to different locations are grouped together over time and form a specific tactile object that are segregated from other tactile stimuli (based on temporal coherence theory). We are not aware of any research about tactile streaming. However, the continuity illusion in auditory perception (i.e. when a portion of the sounds are completely removed and replaced by a loud noise, listeners believe they hear the sound continuously behind the interrupting sounds; see [8] for a review) also exists in tactile perception [9] indicates a general rule for perceptual grouping over time in auditory and tactile perception.

Temporal coherence theory is supposed to work not only within but also across modalities. In other words, this theory predicts that temporally coherent auditory and tactile features can be grouped over time and form a specific perceptual object that can be segregated from other auditory or tactile features. Previous studies have shown that features from different modalities can be grouped together if they are temporally contingent [10], [11], [12], [13]. In addition, since the auditory and somatosensory systems both have receptors that can detect mechanical stress generated by vibrating sources in the environment, it is not surprising that several previous studies have found that auditory perception and tactile perception interact with each other at several sensory processing levels. For example, auditory stimuli have been found to bias frequency perception of tactile stimuli that are applied simultaneously [14]. The trigeminal nerve has been found to innervate the dorsal cochlear nucleus (DCN) in cats [15], [16], [17]. The primary auditory cortex has been found to be activated and modulated by tactile stimuli [18], [19]. Moreover, auditory streaming perception has been found to be related to enhanced neural activities in the intraparietal sulcus, which is suggested to be involved in multi-sensory integration [20].

Although previous studies support the hypothesis that temporally contingent auditory and tactile stimuli can be grouped together, it is not clear whether this crossmodal grouping can be streamed over time. The answer to this question can help us clarify where perceptual grouping over time takes place in information processing. For example, previous studies show that auditory stimuli with the same spectral information but different pitches can be segregated into different streams [3], [21]. This observation suggests that auditory stream segregation can take place in or above the processing level for pitch perception. The following fMRI and MEG experiments showed that increasing stream segregation due to large fundamental frequency difference is correlated with increasing activation in the primary and surrounding non-primary auditory cortex [21]. In the present study, if the features in different modalities can be grouped over time, this finding would indicate that the processing level for perceptual grouping over time might take place in or above the processing level for multi-sensory integration. On the other hand, if features in different modalities cannot be grouped over time, this finding would indicate that the processing level for perceptual grouping over time might take place below the processing level for multi-sensory integration.

**Figure 1:**
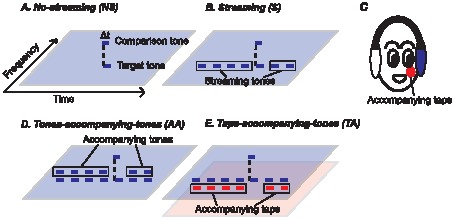
Auditory experimental conditions. The blue lines represent pure tones at different frequencies sent to the left ear (see the blue oval in (C)). The red lines represent tactile stimuli sent to the left cheek (see the red oval in (C)). (A, B, D, E) are the ‘no-streaming’ (NS) condition, ‘streaming’ (S) condition, ‘tones-accompanying-tones’ (AA: auditory-on-auditory) condition, and ‘taps-accompanying-tones’ (TA: tactile-on-auditory) condition.

**Figure 2:**
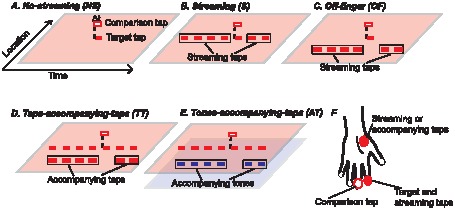
Tactile experimental conditions. The red lines with different patterns represent tactile stimuli applied to different locations on the left hand (see (F)). The blue lines represent auditory stimuli sent to the left ear. (A–E) are the ‘no-streaming’ (NS) condition, ‘streaming’ (S) condition, ‘off-frequency’ (OF) condition, ‘taps-accompanying-taps’ (TT: tactile-on-tactile) condition, and ‘tones-accompanying-taps’ (AT: auditory-on-tactile) condition.

In our experiment, to measure the formation of perceptual stream objectively, we used synchrony detection as a probe for observing streaming formation [22]. The previous study found that the threshold of synchrony detection of a pair of pure tones at different frequencies increases when one of the pure tones is embedded in an auditory stream with pure tones at the same frequency (compare Figure 1A and 1B). Since judging the temporal relationship of the sound events becomes difficult when they are in different auditory streams [3], [22], the degradation of temporal relationship judgment is proposed for probing auditory stream formation [22]. Here we applied this objective measurement to perceptual grouping over time not only in auditory perception but also in tactile perception (Figure 2A and 2B). For example, in the ‘off-frequency’ condition in the previous study [22], one of the pure tones (for synchrony detection) is lagged and preceded by pure tones at different frequency. It is argued that the pure tones at different frequencies do not form an auditory stream, so the threshold of synchrony detection does not increase. The present study had a similar condition in tactile perception, the ‘off-finger’ condition, where one of the taps (for synchrony detection) is lagged and preceded by taps applied on different fingers (Figure 2C). The results in this condition would help us clarify if tactile stimuli are segregated into different perceptual streams if they are applied to different locations.

## Methods

### Ethics Statement

Before the experiment, participants were provided with an information sheet which outlined the general purpose of the study and informed them that they could withdraw at any time without penalty. All participants except the author signed the consent form. All methods employed in this study were approved by the ethics committee in NTT Communication Science Laboratories, and were in accordance with the Declaration of Helsinki.

### Participants

Seven participants (four females), between the ages of 26 and 35 years old, took part in the experiment. One of the participants was the author (IL). All participants had normal hearing, defined as pure-tone hearing thresholds of 15 dB HL or less at audiometric frequencies between 125 and 8000 Hz. No participants was found to have somatosensory deficit or abnormality. All participants (except the author IL) were compensated at an hourly rate.

### Apparatus

The experiment was conducted in a sound-insulated booth. Auditory and tactile stimuli were generated by MATLAB (7.10.0). They were presented with an audio interface (M-AUDIO FAST FIREWIRE 410), which provided five output channels for the auditory stimuli (two channels) and tactile stimuli (three channels). The auditory stimuli were sent to the participants through the audio interface to the headphones (Senheiser HDA200). The tactile stimuli were generated by three vibration generators (Attachable Speaker, Eishindenki). When the tactile stimulus was sent to participants’ left cheek, participants put their head on a chin-rest while the vibration generator was set between the chin-rest and the participants’ cheek. There was a cushion between the vibration generator and the chin-rest so that vibration was not transmitted to the table. When the tactile stimuli were sent to participants’ left hand, the vibration generators were fixed on the palmar side of the index and middle finger tips with velcro tape. Participants put their left hand on their left thigh with the palmar side down under a table so that the transmission of the sounds from the vibration generators to participants’ ears was minimized. There was a cushion between the vibration generator and participants’ left thigh so that participants would not feel the vibration on their thigh when the tactile stimuli were sent to participants’ left thenar eminence.

### Conditions

The experiment was divided into two parts: perceptual grouping over time in the auditory modality (Figure 1) and in the tactile modality (Figure 2). The experimental paradigm was similar to that used in a previous study [22]. Subjects were asked to judge whether a pair of pure tones at different frequencies (in the auditory experiment) or a pair of taps applied to different fingers (in the tactile experiment) were synchronous. The target tone or the target tap (in the pair for synchrony detection) could be presented alone (in the ‘no-streaming’ condition; see Figure 1A for the auditory experiment and Figure 2A for the tactile experiment) or with the streaming tones/taps at the same frequency/location (in the ‘streaming’ condition; see Figure 1B for the auditory experiment and Figure 2B for the tactile experiment). In the case of auditory streaming, the previous study [22] showed that the threshold of synchrony detection is higher in the ‘streaming’ condition than in the ‘no-streaming’ condition. In other words, the streaming process degrades the temporal resolution of the target tone. They also showed that when the target tone is presented with the streaming tones at a different frequency or to a different ear (in the ‘off-frequency’ and ‘off-ear’ conditions; not tested in the present study), the thresholds of synchrony detection in these conditions are similar to the threshold in the ‘no-streaming’ condition. In other words, when the target is ‘released’ from the streaming process, the temporal resolution of the target is preserved. In the present study, we investigated whether the target tap is released from the streaming process when the streaming taps are applied to another location (in the ‘off-finger’ condition; Figure 2C). The ‘off-finger’ taps were applied to the left thenar eminence (the red circle with slashes in Figure 2F).

To investigate whether temporal coherence theory can be applied to perceptual grouping over time within the auditory and tactile modalities, we added the tones/taps accompanying the streaming tones/taps (‘tones-accompanying-tones’ and ‘taps-accompanying-taps’ conditions; see Figure 1D and 2D, respectively). If the accompanying tones/taps ‘capture’ the streaming tones/taps and release the target tone/tap from the streaming process, the thresholds of synchrony detection in these conditions should be similar to the threshold in the ‘no-streaming’ condition.

To investigate whether temporal coherence theory can be applied to perceptual grouping over time across the auditory and tactile modalities, we added the taps/tones accompanying the streaming tones/taps (in the ‘taps-accompanying-tones’ and ‘tones-accompanying-taps’ conditions; see Figure 1E and 2E, respectively). If the accompanying taps/tones ‘capture’ the streaming tones/taps and release the target tone/tap from the streaming process (in another modality), the thresholds of synchrony detection in these conditions should be similar to the threshold in the ‘no-streaming’ condition. A piloting auditory experiment had been done when the tactile stimuli were applied to the left hand (in the ‘taps-accompanying-tones’ condition), and no effect of such tactile stimuli on auditory streaming was found. In the present experiment, to make the accompanying taps close to the sound source in the ‘taps-accompanying-tones’ condition, the taps were applied to participants’ left cheek (the red oval in Figure 1C) while the sounds were sent to the participants’ left ear (the blue oval in Figure 1C). Loud noise was sent to the participants through the earphone to mask the sounds generated by the tactile generator.

All participants performed the auditory experiment first, and then performed the tactile experiment. Each experiment started with one adaptive track for each condition as training. If the participant found the task difficult to understand or his/her threshold was measured to be around the ceiling level (see the ‘Procedure’ section), a second adaptive track was conducted to make the participant familiar with the task. After the training, there were four blocks in each experiment, and each block contained one adaptive track for each condition. The order of the adaptive tracks for each condition in each block was randomized. Participants took a break (15 to 20 minutes) when they had performed three adaptive tracks (for about 15 to 20 minutes). The whole experiment took around eight hours. Each participant visited our lab for two days to finish the experiment.

### Stimuli

In the experiment for auditory perception, the frequency of the target tone and the streaming tones was 400 Hz, the frequency of the comparison tone (for synchrony detection) was 755 Hz (11 semitones above the target tone), and the frequency of the accompanying tones was 599 Hz (7 semitones above the target tone). All pure tones were 60-ms long (with 10-ms ramps). The target tone and the streaming tones were separated from each other by 60-ms silent gaps. In the ‘taps-accompanying-tones’ condition, the tactile stimuli were also 60-ms long (with 10-ms ramps), and they were separated from each other and the target tone by 60 ms. The frequency of these tactile stimuli was 200 Hz. The accompanying tones/taps and the streaming tones had the same onset and offset. All of the pure tones described above were sent to participants’ left ear only. The tactile stimuli were applied to participants’ left index finger tip (palmar side).

In the experiment for tactile perception, the frequency of the tactile stimuli was 200 Hz. All the tactile stimuli were 75-ms long (with 10-ms ramps). The target tap and the streaming taps were separated from each other by 75-ms silent gaps. All tactile stimuli were applied to the left hand. The target tap was always applied to the left index finger tip (palmar side; the red oval in Figure 2F). The comparison tap was always applied to the left middle finger tip (palmar side; the red circle in Figure 2F). The streaming taps were either applied to the left index finger tip (in the ‘streaming,’ ‘taps-accompanying-taps,’ and ‘tones-accompanying-taps’ conditions; the red oval in Figure 2F) or the thenar eminence (in the ‘off-finger’ condition; the red circle with slashes in Figure 2F). In the ‘taps-accompanying-taps’ condition, the accompanying taps were applied to the thenar eminence (the red circle with slashes in Figure 2F). In the ‘tones-accompanying-taps’ condition, the pure tones were also 75-ms long (with 10-ms ramps), and they were separated from each other and the target tap by 75 ms. The frequency of these pure tones was 100 Hz. The accompanying tones/taps and the streaming taps had the same onset and offset. The pure tones were sent to participants’ left ear through earphones.

Because the tactile stimuli were audible, low-pass white noise (generated on each trial, with a cut-off frequency at 350 Hz) was sent to both participants’ ears to mask the sounds generated by the vibration generators. In the auditory experiment, the masking noise was 73 dB SPL, while the target tone and the streaming tones were 73 dB SPL, the comparison tone was 74 dB SPL, and the accompanying tones were 68 dB SPL. In the tactile experiment, the masking noise was 68 dB SPL, and the accompanying tones were 70 dB SPL. The masking noise was louder in the auditory experiment than in the tactile experiment because the accompanying taps in the auditory experiment were applied to the cheeks, and the masking noise must be loud enough to mask the sounds generated by the vibration generator that might be transmitted to the ears through bone conduction.

### Procedure

Each trial consisted of two intervals, separated by a 500-ms silent gap. In one of the two intervals, chosen at random to be the first or the second one with equal probability, the target and the comparison tone/tap were presented simultaneously; in another interval, the target was either lagged or preceded the comparison tone/tap (with equal probability) by a time delay, Δt (see Figure 1A and 2A for example). Listeners were instructed to select the interval in which the target and comparison tone/tap were presented simultaneously. After listeners gave their answer, feedback was provided in the form of a message on the computer screen (‘correct’ or ‘incorrect’).

The threshold of synchrony detection was measured by using an adaptive staircase procedure with a 2-down 1-up rule, which tracks the point corresponding to 70.7% correct on the psychometric function [23]. A total of four adaptive tracks, with a minimum of 50 trials and 9 reversals each, were obtained for every condition. In the beginning of each adaptive track, Δt was set to 60 ms in the auditory experiment and 75 ms in the tactile experiment. After two consecutive correct responses or one incorrect response, the value of Δt was decreased or increased, respectively, by a factor of √2. An even number of reversals, beginning with the fourth or fifth, were averaged to obtain one threshold estimate. During each adaptive track, the value of Δt was not allowed to exceed the initial value of Δt. For example, in the auditory experiment, if the adaptive staircase procedure called for a value of Δt greater than 60 ms, Δt was set to 60 ms and the procedure continued.

## Results

**Figure 3:**
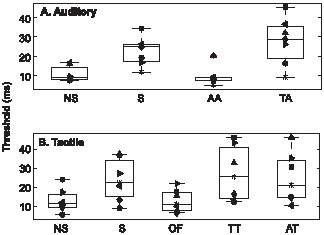
Synchrony detection in the auditory and tactile experiments. (A) The averaged thresholds in different conditions in the auditory experiment. (B) The averaged thresholds in different conditions in the tactile experiment. NS represents the ‘no-streaming’ condition, S represents the ‘streaming’ condition, AA represents the ‘tones-accompanying-tones’ (auditory-on-auditory) condition, TA represents the ‘taps-accompanying-tones’ (tactile-on-auditory) condition, OF represents the ‘off-finger’ condition, TT represents the ‘taps-accompanying-taps’ (tactile-on-tactile) condition, and AT represents the ‘tones-accompanying-taps’ (auditory-on-tactile) condition. Different symbols represent individual participants, and the box plots show the median and 25% and 75% quartiles of the distributions.

For each listener and each condition, thresholds measured in the four adaptive tracks were averaged. Consistent with the use of a geometric tracking rule in the adaptive procedure, geometric means were used when averaging the thresholds across the four adaptive tracks. The statistical analyses, paired t-test (two-tailed), were also performed on log-transformed thresholds. The individual thresholds in each condition and the boxplot for the distribution across participants were shown in Figure 3A (for the auditory experiment) and 3B (for the tactile experiment).

For each experiment described below, the threshold in the ‘no-streaming’ condition was considered to be the baseline for the target tone/tap’s being released from the streaming process, and the threshold in the ‘streaming’ condition was considered to be the baseline for the target tone/tap’s not being released from the stream process. In other words, if the threshold in a test condition was significantly different from the threshold in the ‘streaming’ condition and not significantly different from the threshold in the ‘no-streaming’ condition, the target tone/tap was considered to be released from the streaming process in that test condition, and vice versa.

In both the auditory and tactile experiments, the threshold of synchrony detection was larger in the ‘streaming’ (S) condition than in the ‘no-streaming’ (NS) condition (p = 0.002 in both experiments). This observation indicates that in the somatosensory system, as in the auditory system, temporal resolution of a target tap was degraded while this target tap was grouped perceptually over time with other streaming taps applied to the same location.

In the auditory experiment, the threshold in the ‘tones-accompanying-tones’ (AA) condition was smaller than the threshold in the ‘streaming’ (S) condition (p<0.001) but similar to the threshold in the ‘no-streaming’ condition (p = 0.14), indicating that accompanying tones captured the streaming tones and then released the target tone from the streaming process. On the other hand, the threshold in the ‘taps-accompanying-tones’ (TA) condition was larger than the threshold in the ‘no-streaming’ (NS) condition (p = 0.002) but similar to the ‘streaming’ (S) condition (p = 0.19), indicating that accompanying taps neither captured the streaming tones nor released the target tone from the streaming process.

In the tactile experiment, the threshold in the ‘off-finger’ (OF) condition was smaller than the threshold in the ‘streaming’ (S) condition (p<0.001) but similar to the threshold in the ‘no-streaming’ (NS) condition (p = 0.99), indicating that tactile stimuli applied to different locations were not grouped perceptually over time. On the other hand, the thresholds in the ‘taps-accompanying-taps’ (TT) and ‘tones-accompanying-taps’ (AT) conditions were larger than the threshold in the ‘no-streaming’ (NS) condition (p<0.001 and 0.02, respectively) but similar to the threshold in the ‘streaming’ (S) conditions (p = 0.3 and 0.91, respectively), indicating that neither accompanying taps nor accompanying tones changed how the target tap was grouped with streaming taps over time.

## Discussion

This study investigated how tactile stimuli are streamed over time and segregated into different streams on the basis of population-separation theory and temporal coherence theory and whether temporal coherent theory can be applied to perceptual grouping over time within and across auditory and tactile modalities.

### Tactile Streaming

The tactile stimuli applied to the same location were found to be grouped over time, and such a grouping process degraded the temporal resolution of the tactile stimulus embedded in the tactile stream. Similar to auditory streaming, tactile stimuli that were applied to different locations were likely to be segregated into different perceptual streams.

In auditory streaming, stimulus-specific suppression is hypothesized to be one of the general mechanisms for segregation of neural activity between two populations of neurons coding for auditory stimuli with different properties [4], [21], [24]. Stimulus-specific suppression may also play an important role in tactile streaming observed in this study. Similar to auditory masking, a tactile stimulus has been found to be masked by another tactile stimulus presented 15 to 100 ms before or 40 to 100 ms after it [25], [26]. In this study, tactile stimuli were presented repeatedly with a 75-ms inter-stimulus interval, so the neurons responding to the tactile stimuli applied to the same location were likely to be suppressed. Tactile adaptation due to neural suppression may be responsible for tactile streaming and the degradation of the temporal resolution of the tactile stimulus that is embedded in a tactile stream.

Auditory stimuli can be segregated by different acoustic properties such as frequency, pitch, timbre, and location. For tactile streaming, we found that stimulus location is an important feature for stream segregation. In the ascending pathway of the somatosensory system, neurons activated by tactile stimuli applied to the index finger tip and those activated by tactile stimuli applied to the thenar eminence are usually not overlapped [27], [28], [29], [30]. Since the tactile stimuli applied to the index finger tip and the thenar eminence activate different groups of neurons, the adaptation of neurons responding to the thenar eminence is not likely to affect the neurons responding to the index finger tip. Therefore, tactile stimuli applied to these two locations were segregated into two perceptual streams, and the tactile stimuli applied to the thenar eminence did not degrade the temporal resolution of the tactile stimulus applied to the index finger tip.

In auditory streaming, the segregation of auditory stimuli is found to depend on frequency separation [2], [6] and spatial separation [31], [32]. For frequency separation, the larger the separation is, the more frequently the auditory stimuli are perceived as separated streams. The present study did not test different spatial arrangements of tactile stimuli, so it is not known if tactile stream segregation depends on spatial distance between the tactile stimuli in a spatiotopic or a somatotopic map. This is an interesting question for future studies.

### Temporal Coherence Theory for Within-modality Streaming

According to temporal coherence theory, features in the same modality can be grouped together over time if they are temporally coherent [1]. In the present study, cross-frequency grouping of the streaming tones and the accompanying tones were found to form a united perceptual stream and then release the target tone from the streaming process, but repeated dual-point simultaneous tactile stimuli did not form a perceptual stream to release the target tap from the streaming process.

Our observation that pure tones with and without temporally coherent accompanying tones were segregated into two streams is consistent with previous studies of rhythmic masking release [33], [34] and of synchrony detection across frequencies in an auditory stream [7]. On the other hand, the temporally coherent streaming taps and accompanying taps that were applied to different locations in our experiment seem to be coded as two percepts instead of forming a united percept. This observation in the tactile experiment should be compared to the tactile funneling illusion. The tactile funneling illusion shows that tactile stimuli simultaneously applied to two locations form a united percept and are localized as somewhere between the true loci [35]. This illusion has been found to be most prominent when the tactile stimuli are applied to the arm, and it is also observed when the tactile stimuli are applied to the index and middle fingers [36], [37], [38]. In this study, the tactile stimuli were applied to the index finger tip and the thenar eminence. The spatial separation between the index finger tip and the thenar eminence may be too large to generate the funneling illusion. According to the subjective reports in the tactile experiment, even when a fused tactile perception between the index finger and thenar eminance was felt, this fused percept was much fainter than the tactile perception at the true stimulus locations.

Based on the present study, we cannot rule out the possibility that simultaneous tactile stimulation on locations nearby (e.g., on the index finger and the middle finger) can produce united percept and be streamed over time. However, this finding reveals an interesting contrast between auditory and tactile perception. In auditory perception, when two tones are presented simultaneously, they are grouped together even when their frequencies are as distant as 1.25 octaves [7]. On the other hand, when two tactile stimuli are presented simultaneously, they are not grouped together even when the spatial distance is as small as the distance between the index finger tip and the thenar eminence.

### Temporal Coherence Theory for Crossmodal Streaming

Temporal coherence theory suggests the features in different modalities might be grouped together over time if they are temporally coherent [1]. In the present study, we did not find that the streaming tones/taps and the accompanying taps/tones form a united perceptual stream and release the target tone/tap from the streaming process. Although temporally coherent auditory stimuli at different frequencies formed a united percept that could be grouped over time, temporally coherent tactile stimuli that were applied to different locations did not. Neither did temporally coherent auditory stimuli and tactile stimuli form a united percept that could be grouped over time when within-modality streaming existed.

The limitation of temporal coherence theory observed here makes sense from an ecological point of view. In the auditory domain, it is highly probable that frequency components produced by a single acoustic event covary together, and those produced by independent acoustic events change independently. On the other hand, this is not always the case in the tactile domain, because one may touch a single object asynchronously with different body parts. For example, when one grabs a cup, her or his palm may touch the cup first, followed by the fingers. As is evident from this example, the timing of tactile stimulation is not solely determined by external objects or events, but depends critically on the way of touching. Moreover, even different objects can produce synchronous tactile stimulation. For example, one can touch an object with the right hand and another object with the left hand at the same time. Therefore, temporal coherence cannot provide a reliable cue for binding in the tactile domain. Similarly, because it is common in a natural environment that one only hear sounds without perceiving any vibration, temporal coherence cannot be a strong cue for audio-tactile binding.

Even if the limitation of temporal coherence theory can be explained from the ecological viewpoint, it is still possible that attention and training will help us bind temporally coherent features in these two modalities together and form a united percept. In many studies that show crossmodal binding, participants either receive extensive training [39], [40] or are instructed to pay attention to both modalities [41]. In some studies that show crossmodal binding without training or attention, the researchers used linguistic materials [42], which we have been exposed to repeatedly in our daily life. In the present study, participants did not receive extensive training, and they were instructed not to pay attention to the modality that was not related to synchrony detection task. The lack of attention and training may be a part of reasons why crossmodal binding was not observed in the present experiment.

Our findings may indicate that the streaming process takes place below audio-tactile integration. Although auditory perception and tactile perception have been found to interact with each other at the peripheral level in several previous studies, the nature of audio-tactile interaction is not clear. For example, although the trigeminal nerve innervates the DCN in cats [15], [16], [17], we do not know how the innervations change auditory perception or whether such innervation exists in humans. Moreover, the innervations to the DCN in cats were mainly from the pinna, and the effects are observed mainly when the pinna is pressed or stretched [15]. In the present experiment, the vibrating tactile stimuli were applied to the cheek, and these differences may explain why crossmodal grouping was not observed. In addition, although the primary auditory cortex is found to be activated and modulated by tactile stimuli, this modulation from the somatosensory inputs may only reset the phase of ongoing neuronal oscillations to amplify neural responses for the arrival sounds [18], [19]. In other words, this audio-tactile interaction is likely to time the inputs to the auditory cortex better and help us better to hear instead of forming a new perceptual object.

Even if the crossmodal interaction at the low level of sensory processing forms a united percept and affects perceptual grouping, since the crossmodal stream and within-modality stream coexist in the present study, these two streaming processes may compete with each other. For example, an auditory stimulus can bias our visual bouncing illusion if it is presented when the two visual disks overlap on the computer screen [10]. However, this (crossmodal) modulation of the auditory stimulus disappears when the auditory stimulus is embedded in an auditory stream [43]. In addition, the sensitivity of visual temporal order judgments can be significantly enhanced by the asynchronous presentation of a pair of auditory stimuli, one presented slightly ahead of the first visual stimulus and the other presented slightly after the second visual stimulus [11]. This ‘temporal ventriloquism’ effect is hypothesized to be related to crossmodal binding. However, this effect disappears when the auditory stimuli are embedded in an ongoing rhythmical stream of identical auditory stimuli [12]. These two studies show that even if the crossmodal grouping does exist, it may still lose the competition to within-modality streaming (of more than two stimuli). On top of it, since the streaming tones/taps and the target tone/tap were at the same frequency/location in our experiment, the streaming process within the auditory/tactile modality was very strong and likely to be the dominant one in the competition.

### Conclusion

The present study found that tactile stimuli applied to the same location were grouped together over time, and tactile stimuli applied to different locations were segregated into two perceptual streams. In addition, although temporally coherent auditory stimuli at different frequencies formed a united percept that could be grouped over time, temporally coherent tactile stimuli that were applied to different locations did not. Neither did temporally coherent auditory stimuli and tactile stimuli form a united percept that could be grouped over time when within-modality streaming existed. These findings indicate that population separation theory can be applied to tactile streaming as well as to auditory streaming. However, there might be limitation of temporal coherence theory when it is applied to perceptual grouping over time.
