# Correction: Perceptual Grouping over Time within and across Auditory and Tactile Modalities

**DOI:** 10.1371/annotation/67ea003c-b8eb-4485-b1f2-d3b984931b60

**Published:** 2012-10-15

**Authors:** I-Fan Lin, Makio Kashino

There was an error in the affiliation.

The correction affiliation is: NTT Communication Science Laboratories, NTT Corporation, Atsugi, Kanagawa, Japan. 

